# Enhancing malaria control using *Lagenaria siceraria* and its mediated zinc oxide nanoparticles against the vector *Anopheles stephensi* and its parasite *Plasmodium falciparum*

**DOI:** 10.1038/s41598-020-77854-w

**Published:** 2020-12-09

**Authors:** V. N. Kalpana, Khaloud Mohammed Alarjani, V. Devi Rajeswari

**Affiliations:** 1 Department of Bio-medical Sciences, School of Biosciences and Technology, VIT, Vellore-14, Tamil Nadu India; 2 Department of Botany and Microbiology, College of Science, King Saud University, Riyadh, 11451 Saudi Arabia

**Keywords:** Microbiology, Environmental sciences, Nanoscience and technology

## Abstract

In many developing countries, there are certain health problems faced by the public, one among them is Malaria. This tropical disease is mainly caused by *Plasmodium falciparum*. It is categorized as a disaster to public health, which increases both mortality and morbidity. Numerous drugs are in practice to control this disease and their vectors. Eco-friendly control tools are required to battle against vector of this significant disease. Nanotechnology plays a major role in fighting against malaria. The present paper synthesized Zinc oxide nanoparticles (ZnO NPs) using zinc nitrate via simple green routes with the help of aqueous peel extract of *Lagenaria siceraria *(*L. siceraria*). The synthesized ZnO NPs were characterized by various biophysical methods. Moreover, the extract of *L. siceraria *and their mediated ZnO NPs was experimented against III instar larvae of *An. stephensi*. The impact of the treatment based on ZnO NPs concerning histology and morphology of mosquito larval was further observed. In the normal laboratory environment, the efficiency of predation of *Poeciliareticulata *(*P. reticulata)* against *An. Stephensi *larvae was found to be 44%, whereas in aqueous *L. siceraria *extract and its mediated ZnO NPs contaminated environment, *P. reticulate *showed predation efficiency of about 45.8% and 61.13% against *An. Stephensi *larva. *L. siceraria *synthesized ZnO NPs were examined against the *Plasmodium falciparum* CQ-sensitive strains. The *L. siceraria *extract and its mediated ZnO NPs showed the cytotoxic effects against HeLa cell lines with an IC_50_ value of 62.5 µg/mL. This study concludes that *L. siceraria* peel extract and *L. siceraria* synthesized ZnO NPs represent a valuable green option to fight against malarial vectors and parasites.

## Introduction

The major prevailing and significant protozoan tropical disease is Malaria. However, almost a century of attempts was taken to exterminate Malaria, which remains as a definite target, with reports of million clinical cases widespread every year threatening the life of more than 3 billion people^1,2^. Among the four parasites of Malaria, *Plasmodium falciparum* is the most supreme and pathogenic one, which spreads the disease to humans and also the main cause of the malarial morbidity and mortality in countries of both the tropical and subtropical regions^3^. The world’s two third of the population live in regions where malaria is regularly found and the rate is nearly 200 million every year. In the past 30 years, in spite of so many advancements made to know about the disease better, relatively few anti-malarial drugs were developed. Therefore, to control the plasmodial activity, there is an urgent requirement for inexpensive and effective anti-malarial drugs^4^.

One of the chief practices followed to control the malaria is controlling vector, because at present there is no effective vaccine against Malaria. The use of insecticides in mosquito control has been identified with numerous issues, which include the resistance development in mosquitoes, toxic effects on humans, and toxicity to non-target organisms. Kaushik et al.^5^ stated that such issues spotlight the rapid necessity to create novel insecticides that are safe, biodegradable, accurate to each other and effective. Zofo et al.^6^ claimed that the conventional herbal remedies are the only feasible source for an inexpensive therapy of Malaria. In the past few years, plants are used as a traditional source for developing drugs to cure malarial disease. The chemical constituents like artemisinin and quinine were extracted from the plants and they were used to cure malaria^6,7^. In order to resist the parasitic activities, an alternative drug is required to control the strains which are resistant in nature. Therefore, pharmacological sector uses NPs to overcome this disease because of their increasing attention. Moreover, their fascinating and unusual properties were impacted strongly through its structure, morphology, and size^8,9^. Nanotechnology is an emerging field in which various plant sources have been utilized for the synthesis of NPs^10–21^. Using plants for synthesizing NPs is safe, eco-friendly, inexpensive, and rapid for the therapeutic usage in the humans^22,23^. Palladium (Pd), Platinum (Pt), Zinc (Zn) and Silver (Ag) are the metallic NPs that have the power to control the malarial population existing in the surrounding^24–30^. Among them, ZnO NPs are reported to have extensive applications in biological and pharmacological areas of research. The nontoxic and low-cost production properties make these NPs suitable in the drug research and in the field of agriculture. The physical and chemical properties of ZnO and other metal oxides are enormously applied in biomedical and in some cancer applications. Therefore, the eco-friendly green synthesis methods to synthesize NPs are gaining interest in the scientific community^23,31^.

In addition, these NPs have the antibacterial capacity^31,32^ and cytotoxic activity^33–36^. Plant extract mediated synthesis of ZnO NPs have been carried out recently in many plant species like *Camellia sinensis*^37^, *Ficus benghalensis*^38^, *Punica granatum*^39^, *Trifolium pretense*^36^, *Hibiscus subdariffa*^40^ and *Aloe vera*^41^ etc. However, on the efficacy of the vegetable peels, information is negligible despite the fact that few vegetables are goitrogenic in nature^42,43^. Wang and Ng^44^ stated that Bottle gourd (*Lagenaria siceraria *(Mol.)) belonging to cucurbits family, *Lagenaria siceraria* regularly known as Ghiya or Dudhi is extensively cultured in both tropical and subtropical areas. It has anti-swelling and diuretic effects. Thus, it is considered to be the main food. Roopan et al.^9^ and Anandh et al.^45^ claimed that the extract of *Lagenaria siceraria*is used in the therapy of large varieties of diseases like ascites, beriberi, and anasarca (edema). Further, the plant has been widely used for various treatment purposes that include antibacterial^45^, cytotoxic^46,47^ and anti-malaria activities^48^. However, those studies applied different NPs but not much in ZnO NPs. Therefore the present paper investigated the larvicidal, anti-plasmodial, cytotoxic and predatory efficiency of aqueous peel extract of *Lagenaria siceraria* and its mediated ZnO NPs.

## Materials and methods

### Chemicals and materials

Zinc nitrate, Xylene, Ethanol and all the chemicals required for carrying out the experiment was procured from Sigma-Aldrich. Fresh *L. siceraria *was obtained from Vellore local market, Tamil Nadu, India. Zinc nitrate was obtained from Sigma Aldrich, India. All aqueous solutions were prepared using deionized water. All glass wares were cleaned with chromic acid followed by thorough washing with deionized water and then with acetone for prior use.

### Preparation of *L. siceraria* aqueous extract

The impurities like dust, scum and other kinds of stuff were removed from the gathered *L. siceraria* by first washing it using tap water and then using distilled water. Later, they were peeled cautiously to isolate the epicarp and instantly dried in the shade. The peels were dried to obtain a fine powder. The 10 g of *L. siceraria* powder was measured and fetched in a beaker that already comprises of 100 mL of distilled water. The mixture was boiled for 10 min^49,50^. Whatmann No.1 filter paper was used to filter the obtained extract and a separate flask was used to accumulate the filtrate and stored in the refrigerator for further use.

### Bio-synthesis and characterization of ZnO NPs using *L. siceraria* peel

Zinc nitrate and *L. siceraria *aqueous peel extract were used to amalgamate ZnO NPs. The detailed synthesis and characterization have been reported in our earlier work^50^.

### *An. stephensi* rearing

*An. stephensi* larvae were collected from rice fields and stagnant waters from the nearby areas of Melvisharam (12°56′23″ N, 79°14′23″ E) and identified in Zonal Entomological Research Centre, Vellore (12°55′48″ N, 79°7′48″ E), Tamil Nadu. To start the colony, larvae were kept in plastic trays containing tap water. All the experiments were carried out at 27 ± 2 °C and 75–85% relative humidity under 14:10 light and dark cycles. Larvae were nourished under a diet of dog biscuits, algae and brewer's yeast in 3:1:1 proportion, respectively^34^.

### Larvicidal activity

In this assay, *An*. *Stephensi*III instar larvae were left in a glass beaker for 24 h containing 250 mL of dechlorinated water along with aqueous *L. siceraria *extract (80, 160, 240, 320 and 400 ppm) and its mediated ZnO NPs (30, 60, 90, 120 and 150 ppm). Using distilled water, the control set-up was made. As a result, a number of larval deaths were noticed after exposure of 24 h. The experiment was repeated five times against the *An. Stephensi *III instar larvae^51,52^. The death rate can be calculated by using the following formula.

$${\text{Mortality}} \, \left( {\text{\% }} \right) = \left( {{\text{number}}\,{\text{of}}\,{\text{dead}}\,{\text{individuals}}/{\text{number}}\,{\text{of}}\,{\text{treated}}\,{\text{individuals}}} \right) \times 100$$

### Histopathological and stereomicroscopic analysis

In order to conduct a histopathological study, *An. Stephensi *larvae with the aqueous extract of *L. siceraria *and ZnO NPs were treated for 24 h with 10% buffered formaldehyde and then dehydrated through the solutions of xylene and ethanol (70–100%) and at last they were mounted in paraffin blocks. With the help of glass knives, larval tissues were segmented in the rotary microtome for a thickness of 8 µm. Each cut sections were mounted on the glass slides and stained by eosin and haematoxylin. After this process, under the microscopic light, each section was examined for the histopathological test. Through the stereomicroscope, the collected larval tissues and its damages were observed^53,54^.

### Predation efficiency assays

In this experiment, the predation efficiency of *Poeciliareticulata *(*P. reticulata*) (National Institute of Health Guidelines) against III instar *An. Stephensi *larvae was examined. In each and every single trail, with one *P. reticulate *nearly 150 larvae were introduced in glass beakers containing 250 mL of dechlorinated water treatment. Aqueous *L. siceraria *extracts and its mediated ZnO NPs (i.e. for plant extract and NPs, nearly 1/3 of LC_50_) were calculated against III instar larvae. The experiment was also performed under standard laboratory conditions (especially with no treatment of plant extract and NPs). Control was dechlorinated water and mosquito larvae without *P. reticulata.* Chandramohan et al.^55^ and Murugan et al.^56^ stated that larvae of mosquitoes were exchanged by new ones daily. Each day for about 12 and 24 h every beaker was checked and the consumption of larvae by *P. reticula* was noted. To normalize the tendency of each and every *P. reticula*; each assessed fish was not feeded before 24 h of the testing. With the below stated formula, the predatory efficiency can be calculated:

$${\text{Predatory efficiency}} = \left[ {\left( {{\text{Number of consumed mosquito larva}}/{\text{Number of predators}}} \right)/{\text{Total number of mosquito larva}}} \right] \times 100$$

### Bio-evaluation method

#### In vitro cultivation of *Plasmodium falciparum*

With the help of low-cost standard assay method Malaria SYBR Green I based fluorescence (MSF), the antiplasmodial activity of aqueous extract of *L. siceraria *and its mediated ZnO NPs was assessed against chloroquine-sensitive 3D7 strains of *Plasmodium falciparum.* The *P. falciparum* culture was maintained at *in-vitro* condition on human erythrocytes (blood group O^+ve^) in RPMI-1640 medium (Sigma) amplified with serum of O Rh^+^ (10%), D-glucose at 0.2%, albumax II at 0.5%, 25 mM *N*-2-hydroxyethylpiperazine-*N*-2-ethanesulfonic acid (HEPES buffer) and sodium carbonate of 0.21%^57^.

### Drug dilutions

Chloroquine (CQ) is a stock solution which is formulated in water (milli-Q grade). Accordingly, dimethyl sulfoxide (DMSO) was used for the preparation of the ZnO NPs. To attain the necessary concentrations, the entire stock solutions were later diluted with culture medium.

### In vitro anti-plasmodial assay

The aqueous peel extract of *L. siceraria *and its mediated ZnO NPs (1.56, 3.12, 6.25, 12.5, 25, 50, 100 µg/mL) was evaluated against the Chloroquine sensitive (3D7) strains of *P. falciparum* for conducting the antiplasmodial activity. For conducting the drug screening, SYBR green I-based fluorescence test was set up. The positive control should be maintained at a culture of parasitized blood cells which must be later treated with chloroquine. With fresh red blood cells, and 2% parasitized *P. falciparum* diluted to 2% hematocrit, the negative control was maintained. With fresh red blood cells, 100 µl of *P. falciparum* diluted to 2% of hematocrit was incorporated in the 96 well tissue culture plates. In an atmosphere filled with 5% of air and CO_2_ mixture, the plates were kept in a CO_2_ incubator at 37 °C. After 72 h, a 100 µl of lysis buffer containing 2 × concentration of SYBR Green-I (Invitrogen) was added to it and incubated at 37 °C for 60 min. The plate was analyzed at 530 ± 20 nm of emission and 485 ± 20 nm of excitation for relative fluorescence units using fluorescence plate reader (BIOTEK, FLX800). The fluorescence counts were plotted against the concentration of the drug in dose–response curves^57,58^. With the help of a microscope, the results were validated after 48 h with Giemsa stain and the average percentage of suppressed parasitemia can be calculated using this formula:

$$\begin{aligned} & {\text{Average}}\,{\text{\% }}\,{\text{suppression}}\,{\text{of}}\,{\text{parasitemia}} = {\text{Average}}\,{\text{\% }}\,{\text{parasitemia}}\,{\text{in}}\,{\text{control}} \\ & \quad \quad - {\text{average}}\,{\text{\% }}\,{\text{suppression}}\,{\text{in}}\,{\text{test}} \times {100}\,{\text{Average}}\,{\text{\% }}\,{\text{parasitemia}}\,{\text{in}}\,{\text{control}} \\ \end{aligned}$$

### Data analysis

The antiplasmodial activity of aqueous *L. siceraria *extract and its mediated ZnO NPs was expressed by the percentage growth inhibition. The concentrations causing 90% inhibition of parasite growth (IC_90_) and 50% inhibition of parasite growth (IC_50_) were calculated using the drug concentration–response curves.

### β-Hematin formation assay

The potential *L. siceraria* extract’s antimalarial activity and its mediated ZnO NPs were estimated using Afshar et al.^59^ technique with slight alterations. In short, *L. siceraria* extract and its mediated ZnO NPs with different concentrations (0–2 mg/mL in DMSO) were incubated with 10 mM oleic acid, 1 M HCl, and 3 mM of hematin. The end volume was finely-tuned to 1 mL by mixing sodium acetate buffer (pH5). Later, the samples were protected at 37 °C with constant shaking during the night. During this process, chloroquine diphosphate was applied as a positive control. After that, the samples were centrifuged at 21 °C for 10 min at 14,000 rpm, and the samples were frequently added with 2.5% (w/v) SDS existing in buffered saline in order to purify thehemozoin pellets (usually 3–8 washes). After this process, it was washed with 0.1 M sodium bicarbonate till the removal of supernatant. In the end, clean pellets were dissolved with 1 mL of NaOH, and a UV spectrophotometer was used to measure the absorbance at 400 nm. DMSO was used as a negative control. The outcomes were noted since the heme crystallization/polymerization’s percentage inhibition (I%) was compared towards the positive control (chloroquine) with the help of the given formula:

$$\begin{aligned} & {\text{I}}\,\left( \% \right) = \left( {{\text{AN}}{-}{\text{AA}}} \right) \times 100 \\ & {\text{AN}} \\ \end{aligned}$$

where AN—absorbance of negative control; AA—absorbance of test samples.

### Cytotoxicity activity on HeLa cells using MTT assay

The cell line HeLa was acquired from Pune’s National Centre for Cell Sciences (NCCS). Dulbecco’s Modified Eagle’s Medium (DMEM-Sigma) was used to preserve the cell line by boosting it with penicillin 100 U/mL, streptomycin 100 μg/mL, and 10% of Foetal Bovine Serum (FBS-Sigma). Cells were propagated at 37 °C in a moisturized environment having CO_2_ of 5%.

The cell line HeLa was seeded and propagated in a 96-well plate as 1 × 10^5^ cells approximately in every well and incubated for 24 h. Once the cell reached the confluence, the different concentrations of ZnO NPs were added and kept for incubation for 24 h at 37 °C with a 5% CO_2_ condition. Then the sample was taken out from the well and washed with phosphate-buffered saline maintained at pH 7.4. 100 µl/well (5 mg/mL) of 0.5% 3-(4, 5-diphenyl–tetrazolium bromide (MTT) 5-dimethyl-2-thiazolyl)-2, was added and incubated for next 4 h. 1 mL of DMSO was added in every well after the incubating process. The measurement of the absorbance at 570 nm was done with UV-spectrophotometer while DMSO was kept as a blank set-up. Measurements were performed and the concentration required for a 50% inhibition (IC_50_) was determined graphically. By using the below formula, viability % of the cell can be determined.

$${\text{\% }}\,{\text{Cell}}\,{\text{viability}} = {\text{A570}}\,{\text{of}}\,{\text{treated}}\,{\text{cells}}/{\text{A570}}\,{\text{of}}\,{\text{control}}\,{\text{cells}} \times 100$$

Graphs were plotted using the concentration of the sample in X-axis and cell viability % at Y-axis. Sample control and cell control were included in all assays to fully compare the assessment of cell viability^43^.

### Ethics declaration

Use of experimental animals, and human participants. This is to confirm that all methods were carried out in accordance with WHO guidelines and Regulations. The fish (*P. reticulata*) was handled according to the National Institute of Health Guidelines for the handling and care of experimental animals and the animal utilization protocol was approved by the Institutional Animal Care, VIT, Vellore.

## Results and discussions

### Larvicidal activity

The larvicidal activity of aqueous peel extract of *L. siceraria *and its mediated ZnO NPs against III instar *An*. *Stephensi *larvae is shown in Table 1. For *L. siceraria *aqueous peel extract the LC_50_ values were found to be 261.67 ppm and LC_90_ were found to be 606.49 ppm (Table 1), and LC_50_ for synthesized ZnO NPs were found to be 56.46 ppm, and LC_90_ were found to be 145.89 ppm (Table 1). Bhuvaneswari et al.^60^ observed the activity of larvicidal through biosynthesized Ag NPs of leaf extract of *Belosynapsiskewensis *against the fourth instar of *A. aegypti *(LC_50_ = 84.2; LC_90_ = 117.3 ppm) and *An. Stephensi *(LC_50_ = 78.4; LC_90_ = 144.7 ppm). Shanmugasundaram and Balagurunathan^61^ revealed that the biosynthesized Ag NPs manifested remarkable activity of larvicidal towards *An. subpictus,* a malarial vector (LC50 = 51.34 mg/L) and *Culexquinquefasciatus *(LC_50_ = 48.98 mg/L). Subramaniam et al.^62^ documented that Ag NPs synthesized from the aqueous leaf extract of *Mimusopselengi *were highly effective against larvae and pupae of the malaria vector *An. Stephensi *(LC_50_ ranged from 12.53 to 23.55 ppm) and the arbovirus vector *Aedesalbopictus *(LC_50_ ranged from 11.72 to 21.46 ppm). It is imperative to know their functioning during the consideration of ZnO NPs larvicidal activity. The impact of ZnO NPs and that of the biochemical components of the *An. stephensi* III instar larvae was regulated. Overall, it was revealed that there were changes caused by the tested samples in the normal biochemical components with a decrease or increase in the action in comparison to the control.

**Table 1:** Larvicidal activity of *L. siceraria* extract and its mediated ZnO NPs against III instar *Anopheles stephensi* larvae (after 24 h exposure).

Treatment	Concentrations (ppm)	LC_50_(LC_90_) ppm	95% confidence limit LC_50_(LC_90_)		Regression equation	*x*^2^
			LFL	UFL		
*L. siceraria* aqueous extract	80 160 240 320 400	261.67 (606.49)	230.96 (523.12)	295.55 (750.08)	*y* = 0.973 + 0.004*x*	0.221
*L. siceraria* mediated ZnO NPs	30 60 90 120 150	56.46 (145.89)	44.94 (132.25)	65.59 (165.56)	*y* = 0.809 + 0.014*x*	0.541

### Histopathological and stereomicroscopic analysis

**Figure 1:**
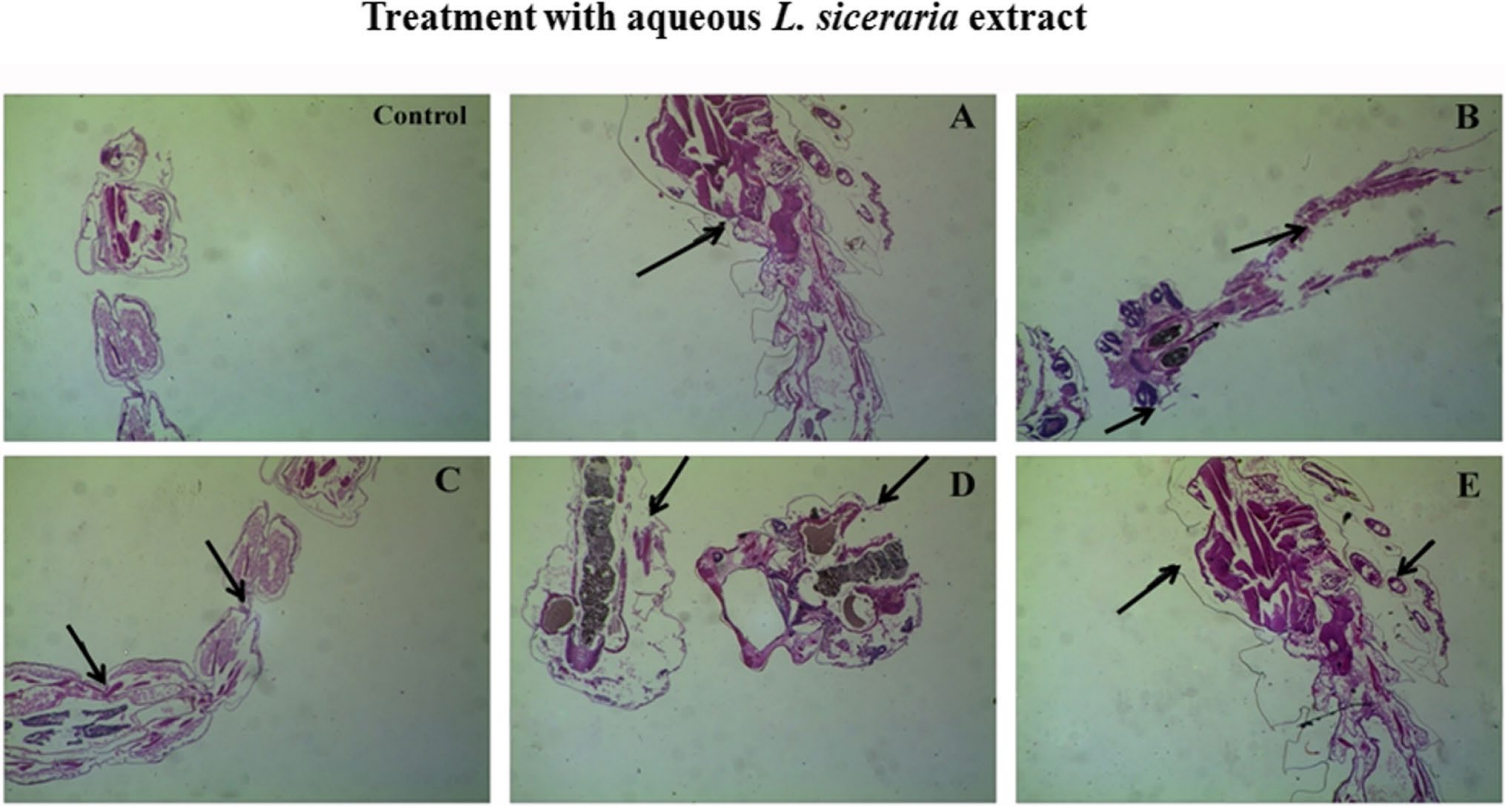
Histopathological images of third instar larvae of *An. Stephensi* treated with: (**A**) 80 ppm of *L. siceraria* aqueous extract. (**B**) 160 ppm *L. siceraria* aqueous extract; (**C**) 240 ppm of *L. siceraria* aqueous extract; (**D**) 320 ppm of *L. siceraria* aqueous extract; (**E**) 400 ppm *L. siceraria* aqueous extract (Arrow indicates disordered and broken epithelial cell, complete break up of midgut and caeca and collapsed larval structure).

**Figure 2:**
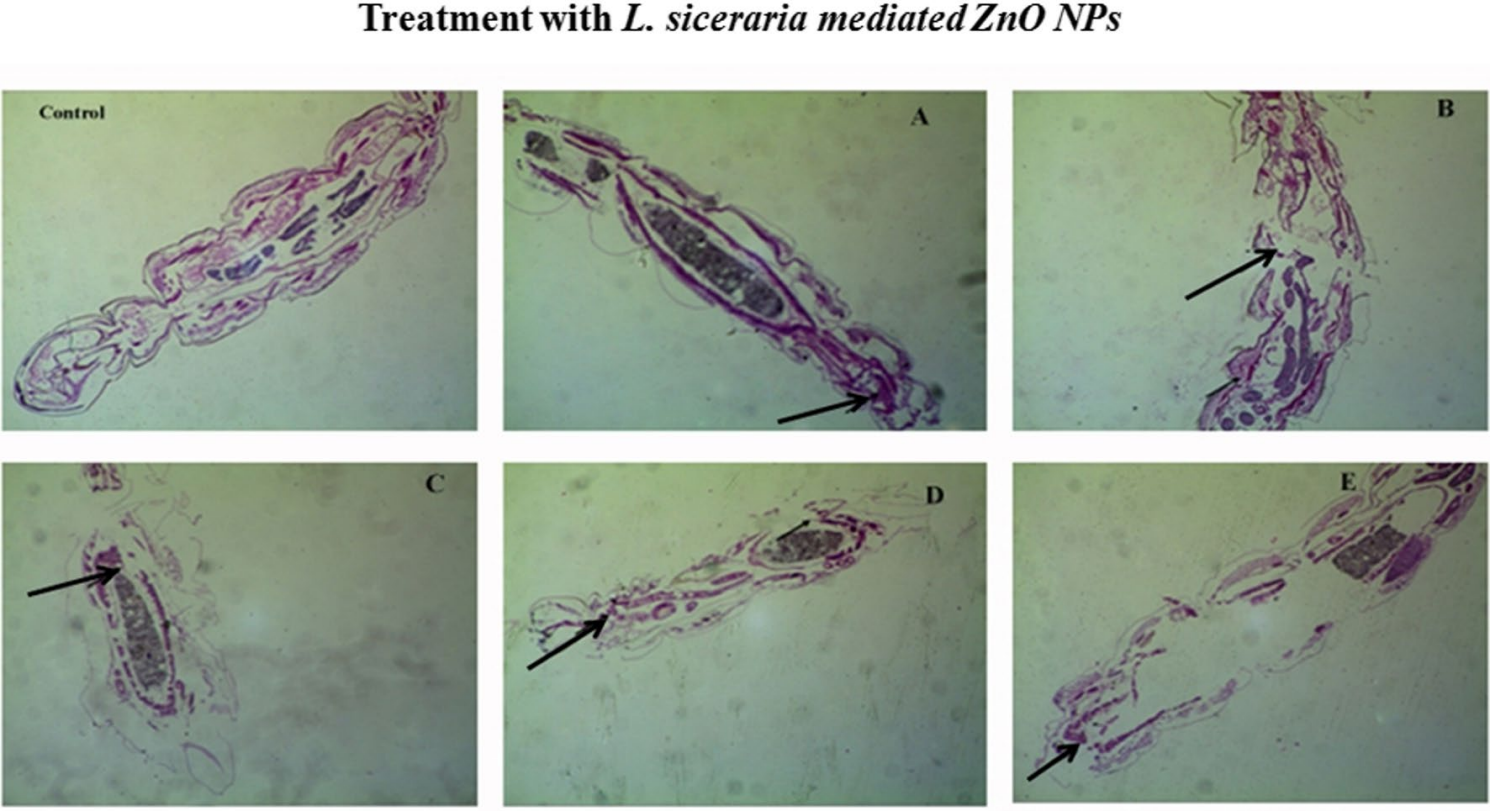
Histopathological images of third instar larvae of *An. Stephensi* treated with: (**A**) 30 ppm of *L. siceraria* mediated ZnO NPs; (**B**) 60 ppm *L. siceraria* mediated ZnO NPs; (**C**) 90 ppm of *L. siceraria* mediated ZnO NPs; (**D**) 120 ppm of *L. siceraria* mediated ZnO NPs; (**E**) 150 ppm *L. siceraria* mediated ZnO NPs (arrow indicates disordered and broken epithelial cell, complete break up of midgut and caeca and collapsed larval structure).

**Figure 3:**
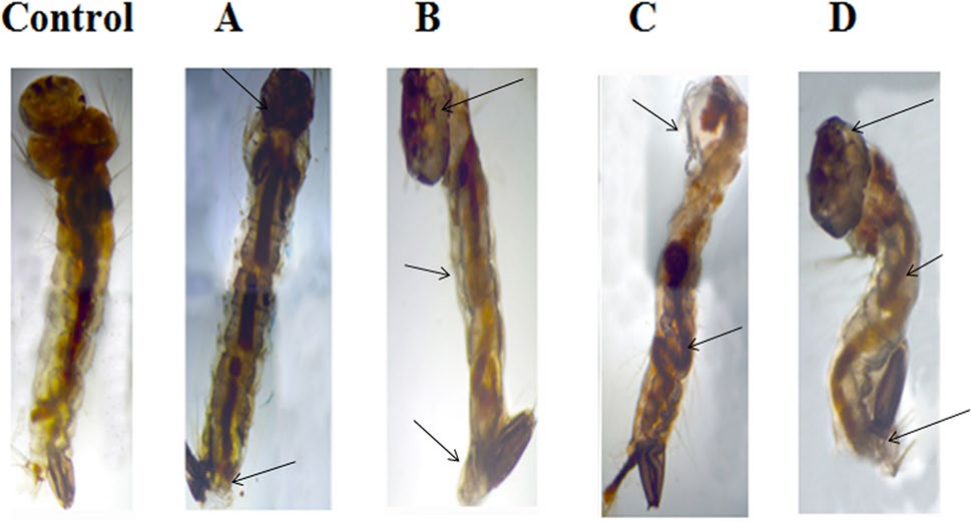
Stereo microscopic image of third instar larvae of *An. Stephensi* treated with; (**A**) LC50 of *L. siceraria* aqueous peel extract (261.67 ppm); (**B**) LC90 of *L. siceraria* aqueous peel extract (606.49 ppm); (**C**) LC50 of *L. siceraria* mediated ZnO NPs (56.46 ppm) (**D)** LC90 of *L. siceraria* mediated ZnO NPs (145.89 ppm) (Arrow indicates damages in the head, abdominal region, thorax region and siphon).

The study of histopathology regarding *An. Stephensi* III instar larvae, where it was made to treat with *L. siceraria* aqueous extract and mediated ZnO NPs, shows the decomposed layer of the epithelial’ s outer cuticle along with entire decomposition of the abdominal area, special caeca, and midgut. This out-turned in the depletion of caudal and lateral hairs (Figs. 1, 2). In the aspect of stereomicroscopic analysis, *An. Stephensi *III instar larvae treated with *L. siceraria *aqueous peel extract and its mediated ZnO NPs represent the decomposed layer of epithelia’s outer cuticle. Also on another side, ZnO NPs mediated by *L. siceraria *represent the depletion of caudal hair, lateral hair, lower head hair, upper head hair, and antenna hair (Fig. 3). The outcomes agree with Ishwarya et al.^51^ who inspected that *A. aegypti* larvae treated with ZnO NPs fabricated by *U. lactuca* represent the disintegration of the cuticle’s outer layer and deposition of Zinc inside the larval body.

### Predation efficiency studies

*P. reticulate *actively predates *An. stephensi *larvae. The predation efficiency of *P. reticulate *towards larvae of *An. Stephensi *was found to be 44% under laboratory conditions. In aqueous *L. siceraria *extract and its mediated ZnO NPs contaminated environment, *P. reticulate *predation efficiency against *An. Stephensi *larvae was found to be 45.8% and 61.13% (Table 2). Haldar et al.^63^ reported that green synthesized NPs will not possess any toxicity towards mosquito natural enemies and predatory fishes.

**Table 2:** Predation efficiency of the *Poecilia reticulata* against III instar larvae of *Anopheles stephensi.*

Treatment	Number of consumed preys		Total predation (n)	Predation (%)
	12 h	24 h		
*L. siceraria*extract	32.6 ± 1.4	36.1 ± 0.2	68.7	45.8
*L. siceraria*mediated ZnO NPs	42.5 ± 0.4	49.2 ± 0.7	91.7	61.13
Standard conditions	31.8 ± 1.0	34.2 ± 0.9	66	44.0

Murugan et al.^56^ stated that *P. reticulata’s *predation towards larvae of *C. quinquefasciatus *had a remarkable increase of predation and there was no notable impact of toxicity accused on guppies as they come into the contact of Ag NPs infected ecosystem. Benelli^64^ found very less toxicity level in non-target organism, *P. reticulata* using green synthesized Ag NPs.

### In vitro antiplasmodial assays

**Figure 4:**
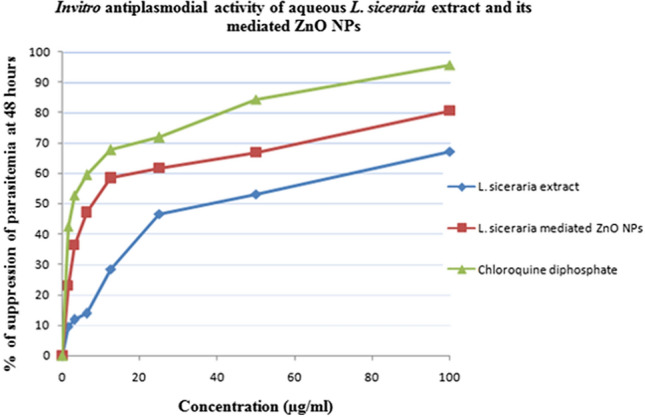
In vitro Antiplasmodial activity of aqueous *L. siceraria* extract and its mediated ZnO NPs.

**Figure 5:**
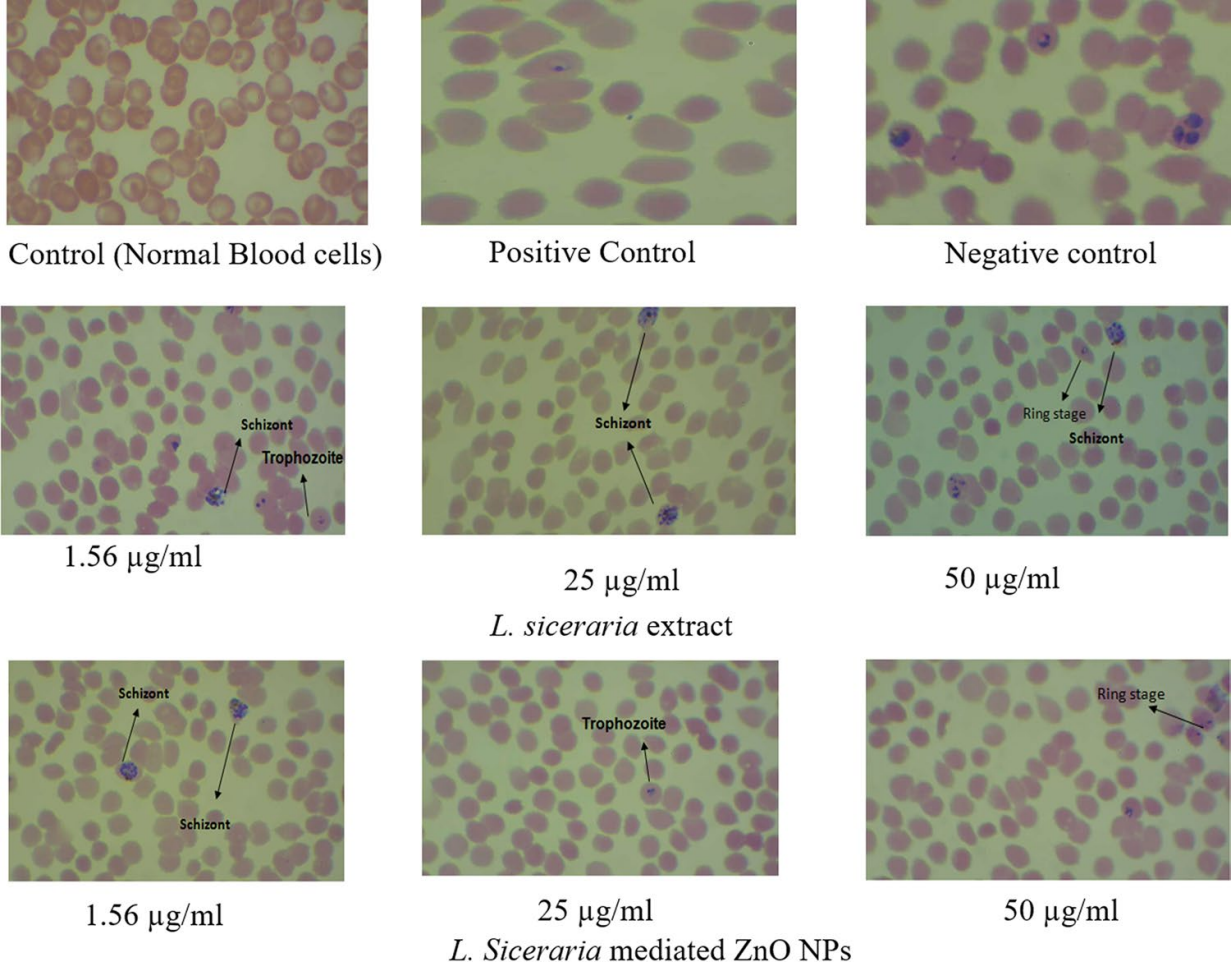
Validation of SYBR Green results by microscopy.

The antiplasmodial activity of aqueous *L. siceraria *extract and its mediated ZnO NPs was tested at a different concentrations ranging from 100, 50, 25, 12.5, 6.25, 3.12, 1.56 µg/mL and chloroquine diphosphate was used as a positive control. The IC_50_ values of aqueous *L. siceraria *extract and its mediated ZnO NPs against *P. falciparum* strains at 48 h of parasitemia suppression are listed in Table 3 and Fig. 4. The microscopic observation involved in anti-plasmodial activity of aqueous extract of *L. siceraria *and its mediated ZnO NPs against *P. falciparum* strains is shown in Fig. 5. In the same way, Mishra and Sharma^39^ noticed that the aqueous extract of leaves of Neem and Ashoka has the property of antiplasmodial at IC_50_ value which is 30 µg/mL and 8 µg/mL. The two medicinally significant plants, namely, *Thalictrumfoliolosum *and *Aristolochiagriffithii *were accessed for in vitro antiplasmodial against *P. falciparum*. The researchers Das et al.^34^ discovered that these medicinal plants are powerful against the resistant and sensitive strains of Chloroquine. The antiplasmodial activity of green synthesized metal oxide and metal NPs were fully studied by Ishwarya et al.^51^.

**Table 3:** In vitro anti-plasmodial activity of aqueous *L. siceraria* extract and its mediated ZnO NPs.

Treatment	% of suppression of parasitemia at 48 h								IC_50_ (µg/ml)	IC_90_ (µg/ml)
	0 µg/ml	1.56 µg/ml	3.12 µg/ml	6.25 µg/ml	12.5 µg/ml	25 µg/ml	50 µg/ml	100 µg/ml		
*L. siceraria*extract	–	9.4 ± 0.7	11.7 ± 0.5	13.8 ± 1.1	28.4 ± 0.9	46.6 ± 1.6	53 ± 2.1	67.1 ± 0.4	14.52	83.07
*L. siceraria* mediated ZnO NPs	–	22.9 ± 0.1	36.2 ± 0.7	47.1 ± 1.2	58.4 ± 0.7	61.6 ± 0.5	66.7 ± 0.3	80.5 ± 0.6	4.32	64.87
chloroquine diphosphate	–	42.5 ± 1.8	52.8 ± 0.6	59.4 ± 2.6	67.6 ± 2.1	71.8 ± 0.8	84.3 ± 1.2	95.6 ± 2.1	2.5	84.53

### β-Hematin formation assay

The outcomes of the formation of cell free β-hematin assay that was carried out on peel extract of *L. siceraria* and ZnO NPs are arranged in Table 4. *L. siceraria* aqueous peel extract showed moderate anti-malarial activity (IC_50_ 2.79 mg/mL), while *L. siceraria* extract mediated ZnO NPs exhibited potent anti-malarial effect with IC_50_ values of 1.38 mg/mL respectively, in comparison to positive control (chloroquine, IC_50_ = 0.91 mg/mL).

**Table 4:** Inhibition of hematin.

Treatment	(% ) inhibition of heme polymerization						IC_50_ (mg/ml)
	0 mg/ml	0.5 mg/ml	1 mg/ml	1.5 mg/ml	2.0 mg/ml	2.5 mg/ml	
*L. siceraria* extract	–	13.1	24.3	32.4	37.4	41.2	2.79
*L. siceraria* mediated ZnO NPs	–	38.9	41.6	53.3	66.5	76.9	1.38
chloroquine diphosphate	–	52.0	64.3	72.2	81.9	94.3	0.91

### The mechanism of ZnO NPs and *L. siceraria* on *P. falciparum* strains

**Figure 6:**
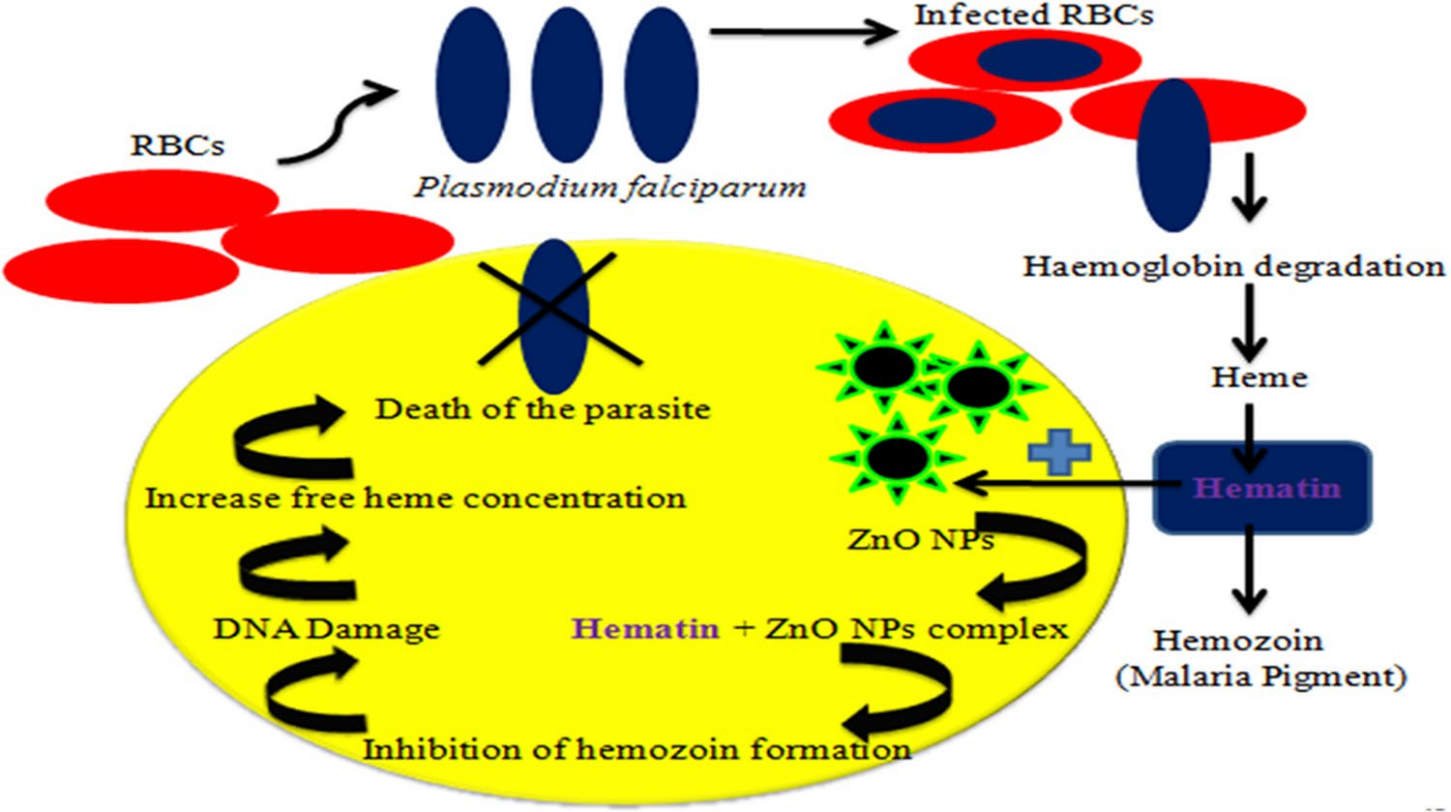
Schematic representation of the mechanism of ZnO NPs on *Plasmodium falciparum*.

The plasmodium parasite outbreaks the host erythrocyte in order to utilize hemoglobin for synthesizing the essential requirements to develop and proliferate. During this process, a massive amount of heme is generated as a toxic undesirable byproduct which is pernicious for malaria parasite. Therefore, to protect itself, the parasite neutralizes large amounts of heme to hemozoin or water-insoluble malaria pigment via the biocrystallization process. Hence, inhibition of hemozoin formation by means of peel extract of *L. siceraria* aqueous mediated ZnO NPs is regarded as an incomparable target to combat the malaria (Fig. 6).

### Cytotoxicity activity on HeLa cells using MTT assay

**Figure 7:**
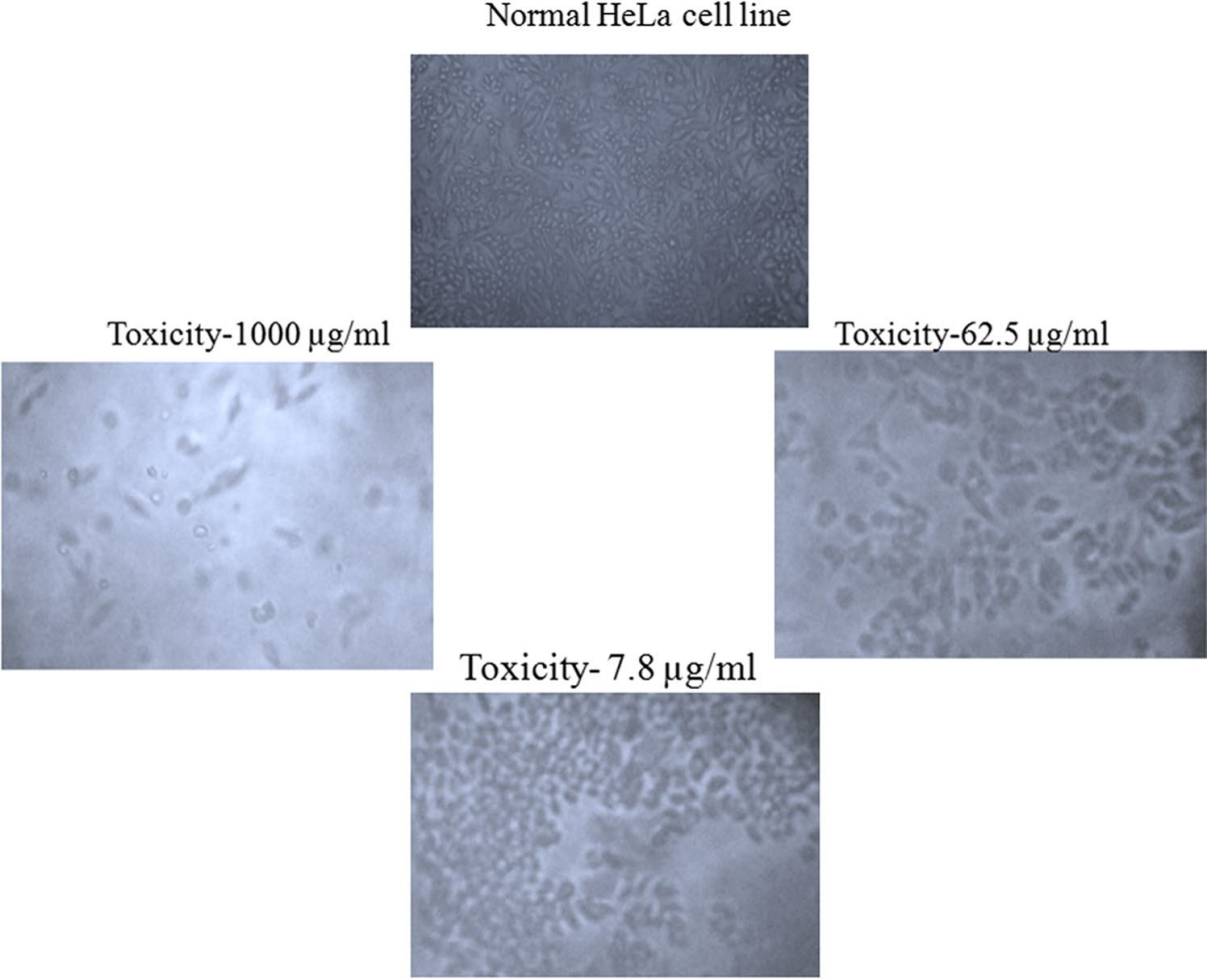
Cytotoxic activity of *L. siceraria* mediated ZnO NPs on HeLa cell line.

**Figure 8:**
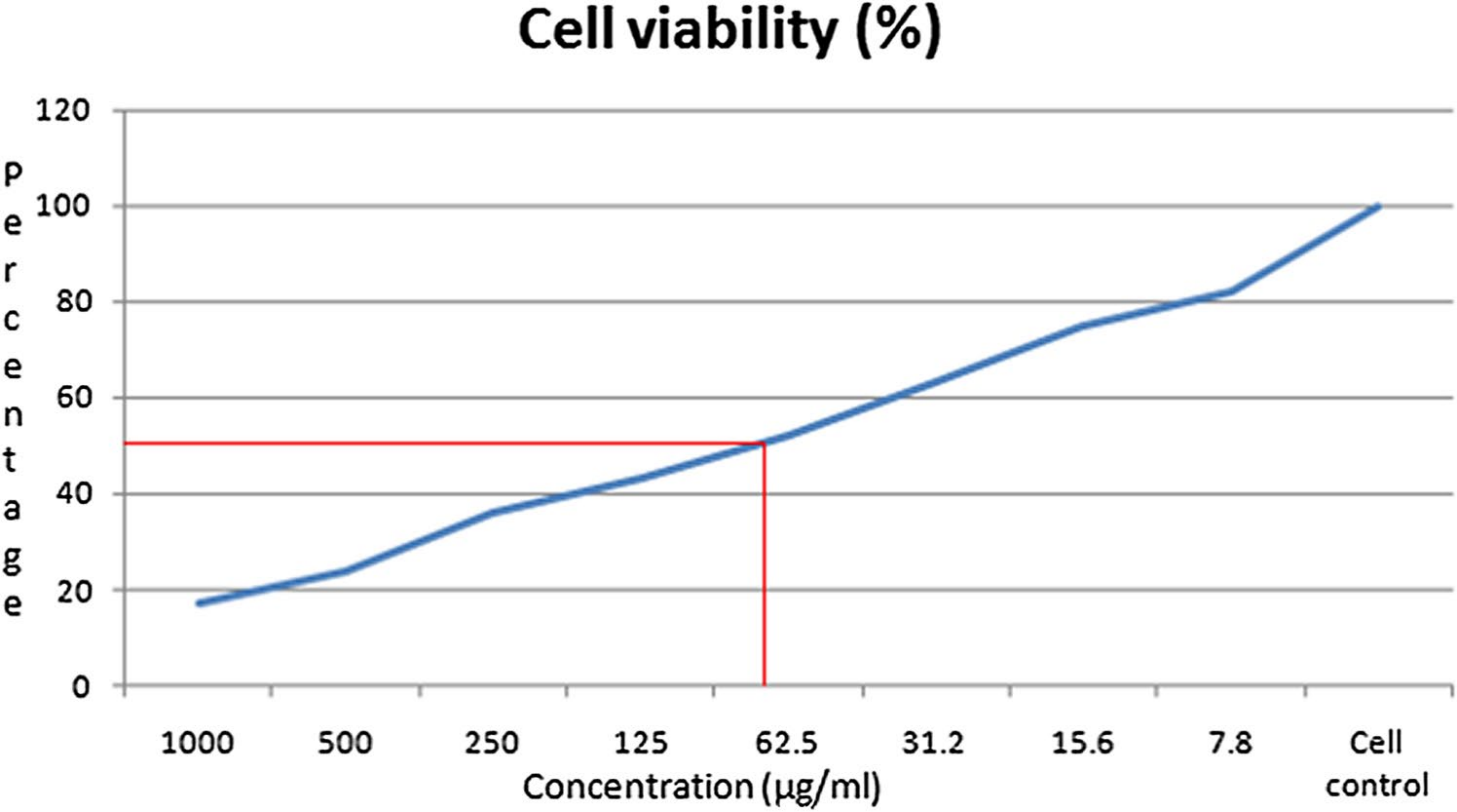
Percentage of inhibition in nanoparticles treated HeLa cells.

The in vitro cytotoxicity of *L. siceraria* mediated ZnO NPs was evaluated against human cervical (HeLa) cancer cell lines at different concentrations (1000, 500, 250, 125, 62.5, 31.2, 15.6, 7.8 µg/mL). The *L. siceraria *mediated ZnO NPs exhibited potent cytotoxicity/anticancer activity in the tested cell lines (Table 5). The effect was compared with normal HeLa cell lines. Results showed that at higher concentrations there is a significant mortality (Fig. 7). The inhibitory effect was observed after 24 h of incubation. Figure 8 shows the changes in the percentage of inhibition in NPs treated HeLa cells. The results also showed that HeLa cells were inhibited by *L. siceraria *mediated ZnO NPs with an IC_50_ value of 62.5 µg/mL (Table 4). Thus, the synthesized NPs were found to be potent cytotoxic agent against HeLa (Cervical cancer) cell line. Similarly, ZnO NPs synthesized from *Abutilonindicum* against HeLa cell lines exhibited potent cytotoxicity towards cell lines of HeLa which was found to have IC_50_ value as 45.82 µg/mL^65^.

**Table 5:** MTT assay test on HeLa cell line after treatment with different concentrations of *L. siceraria* mediated ZnO NPs.

S. no	Concentration (µg/ml)	Dilutions	Absorbance (O.D)	Cell viability (%)
1	1000	Neat	0.184	17.03
2	500	1:1	0.259	23.98
3	250	1:2	0.388	35.92
4	125	1:4	0.469	43.42
5	62.5	1:8	0.562	52.03
6	31.2	1:16	0.684	63.33
7	15.6	1:32	0.810	75.00
8	7.8	1:64	0.889	82.31
9	Cell control	–	1.080	100

## Conclusion

Overall this paper reviews the use of nanomaterials for controlling malaria and mainly examines the malaria life cycle, epidemiology and prevalence in global and India perspective. From this review, it is clear that female anopheles mosquito play a significant role in transmits malarial disease and hence in recent days, many researchers applied various nanotechnology methods to control it specifically nanomimics strategy. However, the application of nanotechnology for controlling malaria has both positive and negative effects but it provides various adverse effects to humans therefore it is recommended to apply for medicinal purpose in future. Nonetheless, further field studies are required to investigate the effective method of behavior and side effects.

## References

[1] Owuor B (2012). In vitro antiplasmodial activity of selected Luo and Kuria medicinal plants. J. Ethnopharmacol..

[2] Benelli G, Mehlhorn H (2016). Declining malaria, rising of dengue and Zika virus: Insights for mosquito vector control. Parasitol. Res..

[3] Passarini GM (2017). In vitro antiplasmodial activity of flower extracts from Combretum leprosum Mart (mofumbo). Ciência e Natura.

[4] Rajakumar G, Rahuman AA (2011). Larvicidal activity of synthesized silver nanoparticles using *Eclipta prostrata* leaf extract against filariasis and malaria vectors. Acta Trop..

[5] Kaushik NK (2015). Evaluation of antiplasmodial activity of medicinal plants from North Indian Buchpora and South Indian Eastern Ghats. Malaria J..

[6] Zofou D, Ntie-Kang F, Sippl W, Efange SM (2013). Bioactive natural products derived from the Central African flora against neglected tropical diseases and HIV. Nat. Prod. Rep..

[7] Gopalakrishnan K, Ramesh C, Ragunathan V, Thamilselvan M (2012). Antibacterial activity of Cu_2_O nanoparticles on *E. coli* synthesized from Tridax procumbens leaf extract and surface coating with polyaniline. Digest J. Nanomater. Biostruct..

[8] Benelli G, Lukehart CM (2017). Applications of green-synthesized nanoparticles in pharmacology, parasitology and entomology. J. Cluster Sci..

[9] Roopan SM, Rajeswari VD, Kalpana V, Elango G (2016). Biotechnology and pharmacological evaluation of Indian vegetable crop *Lagenaria siceraria*: An overview. Appl. Microbiol. Biotechnol..

[10] Munawer U (2020). Biofabrication of gold nanoparticles mediated by the endophytic Cladosporium species: Photodegradation, in vitro anticancer activity and in vivo antitumor studies. Int. J. Pharm..

[11] Samuel MS (2020). Green synthesis of cobalt-oxide nanoparticle using jumbo Muscadine (Vitis rotundifolia): Characterization and photo-catalytic activity of acid Blue-74. J. Photochem. Photobiol. B.

[12] Sunayana N (2020). Chaetomium globosum extract mediated gold nanoparticle synthesis and potent anti-inflammatory activity. Anal. Biochem..

[13] Brindhadevi K (2020). Zinc oxide nanoparticles (ZnONPs)-induced antioxidants and photocatalytic degradation activity from hybrid grape pulp extract (HGPE). Biocatal. Agric. Biotechnol..

[14] Govindaraju K (2020). Unveiling the anticancer and antimycobacterial potentials of bioengineered gold nanoparticles. Process Biochem..

[15] Shanmuganathan R, Sathishkumar G, Brindhadevi K, Pugazhendhi A (2020). Fabrication of naringenin functionalized-Ag/RGO nanocomposites for potential bactericidal effects. J. Mater. Res. Technol..

[16] Gnanasekar S (2020). Chrysin-anchored silver and gold nanoparticle-reduced graphene oxide composites for breast cancer therapy. ACS Appl. Nano Mater..

[17] Sathiyavimal S, Vasantharaj S, Kaliannan T, Pugazhendhi A (2020). Eco-biocompatibility of chitosan coated biosynthesized copper oxide nanocomposite for enhanced industrial (Azo) dye removal from aqueous solution and antibacterial properties. Carbohyd. Polym..

[18] Marimuthu S (2020). Silver nanoparticles in dye effluent treatment: A review on synthesis, treatment methods, mechanisms, photocatalytic degradation, toxic effects and mitigation of toxicity. J. Photochem. Photobiol. B.

[19] Shanmuganathan R (2020). Core/shell nanoparticles: Synthesis, investigation of antimicrobial potential and photocatalytic degradation of Rhodamine B. J. Photochem. Photobiol. B.

[20] Jeyarani S (2020). Biomimetic gold nanoparticles for its cytotoxicity and biocompatibility evidenced by fluorescence-based assays in cancer (MDA-MB-231) and non-cancerous (HEK-293) cells. J. Photochem. Photobiol. B.

[21] Samuel MS, Jose S, Selvarajan E, Mathimani T, Pugazhendhi A (2020). Biosynthesized silver nanoparticles using *Bacillus amyloliquefaciens*; application for cytotoxicity effect on A549 cell line and photocatalytic degradation of p-nitrophenol. J. Photochem. Photobiol. B.

[22] Rajakumar G (2017). Evaluation of anti-cholinesterase, antibacterial and cytotoxic activities of green synthesized silver nanoparticles using from *Millettia pinnata* flower extract. Microbial Pathog..

[23] Rasmussen JW, Martinez E, Louka P, Wingett DG (2010). Zinc oxide nanoparticles for selective destruction of tumor cells and potential for drug delivery applications. Expert Opin. Drug Deliv..

[24] Hariharan D (2019). Green approach synthesis of Pd@TiO_2_ nanoparticles: Characterization, visible light active picric acid degradation and anticancer activity. Process Biochem..

[25] Varadavenkatesan T (2019). Photocatalytic degradation of Rhodamine B by zinc oxide nanoparticles synthesized using the leaf extract of *Cyanometra ramiflora*. J. Photochem. Photobiol. B.

[26] Shanmuganathan R (2019). Synthesis of silver nanoparticles and their biomedical applications—A comprehensive review. Curr. Pharm. Des..

[27] Pugazhendhi A, Prabakar D, Jacob JM, Karuppusamy I, Saratale RG (2018). Synthesis and characterization of silver nanoparticles using *Gelidium amansii* and its antimicrobial property against various pathogenic bacteria. Microbial Pathog..

[28] Sisubalan N (2018). ROS-mediated cytotoxic activity of ZnO and CeO_2_ nanoparticles synthesized using the *Rubia cordifolia* L. leaf extract on MG-63 human osteosarcoma cell lines. Environ. Sci. Pollut. Res..

[29] Pugazhendhi A, Edison TNJI, Karuppusamy I, Kathirvel B (2018). Inorganic nanoparticles: A potential cancer therapy for human welfare. Int. J. Pharm..

[30] Saravanan M, Arokiyaraj S, Lakshmi T, Pugazhendhi A (2018). Synthesis of silver nanoparticles from *Phenerochaete chrysosporium* (MTCC-787) and their antibacterial activity against human pathogenic bacteria. Microb. Pathog..

[31] Xie Y, He Y, Irwin PL, Jin T, Shi X (2011). Antibacterial activity and mechanism of action of zinc oxide nanoparticles against *Campylobacter jejuni*. Appl. Environ. Microbiol..

[32] Sirelkhatim A (2015). Review on zinc oxide nanoparticles: Antibacterial activity and toxicity mechanism. Nano-micro Lett..

[33] Baek, M. *et al.* in *Journal of Physics: Conference Series.* 012044 (IOP Publishing).

[34] Das D, Nath BC, Phukon P, Dolui SK (2013). Synthesis of ZnO nanoparticles and evaluation of antioxidant and cytotoxic activity. Colloids Surf. B.

[35] Prashanth G (2015). In vitro antibacterial and cytotoxicity studies of ZnO nanopowders prepared by combustion assisted facile green synthesis. Karbala Int. J. Mod. Sci..

[36] Dobrucka R, Dlugaszewska J, Kaczmarek M (2018). Cytotoxic and antimicrobial effects of biosynthesized ZnO nanoparticles using of *Chelidonium majus* extract. Biomed. Microdevice.

[37] Shah RK, Boruah F, Parween N (2015). Synthesis and characterization of ZnO nanoparticles using leaf extract of *Camellia sinesis* and evaluation of their antimicrobial efficacy. Int. J. Curr. Microbiol. Appl. Sci..

[38] Shekhawat M, Ravindran C, Manokari M (2015). A green approach to synthesize the zinc oxide nanoparticles using aqueous extracts of *Ficus benghalensis* L. Int. J. BioSci. Agric. Technol..

[39] Mishra V, Sharma R (2015). Green synthesis of zinc oxide nanoparticles using fresh peels extract of *Punica granatum* and its antimicrobial activities. Int. J. Pharma Res. Health Sci..

[40] Bala N (2015). Green synthesis of zinc oxide nanoparticles using *Hibiscus subdariffa* leaf extract: Effect of temperature on synthesis, anti-bacterial activity and anti-diabetic activity. RSC Adv..

[41] Ali K (2016). Aloe vera extract functionalized zinc oxide nanoparticles as nanoantibiotics against multi-drug resistant clinical bacterial isolates. Journal of colloid and interface science.

[42] Nara K, Miyoshi T, Honma T, Koga H (2006). Antioxidative activity of bound-form phenolics in potato peel. Biosci. Biotechnol. Biochem..

[43] Chandra AK, Mukhopadhyay S, Lahari D, Tripathy S (2004). Goitrogenic content of Indian cyanogenic plant food & their in vitro anti-thyroidal activity. Indian J. Med. Res..

[44] Wang H, Ng T (2000). Lagenin, a novel ribosome-inactivating protein with ribonucleolytic activity from bottle gourd (*Lagenaria siceraria*) seeds. Life Sci..

[45] Anandh B, Muthuvel A, Emayavaramban M (2014). Bio synthesis and characterization of silver nanoparticles using *Lagenaria siceraria* leaf extract and their antibacterial activity. Int. Lett. Chem. Phys. Astron..

[46] Chen C-R, Chen H-W, Chang C-I (2008). D: C-Friedooleanane-type triterpenoids from *Lagenaria siceraria* and their cytotoxic activity. Chem. Pharm. Bull..

[47] Ghosh K, Chandra K, Ojha AK, Sarkar S, Islam SS (2009). Structural identification and cytotoxic activity of a polysaccharide from the fruits of *Lagenaria siceraria* (Lau). Carbohyd. Res..

[48] Menpara D, Desai D, Rathod T, Chanda S (2014). Evaluation of nutraceutical bottle gourd (*Lagenaria siceraria*) as a potential source of natural antimicrobial agent. Am. J. Phytomed. Clin. Ther..

[49] Kalpana V, Payel C, Rajeswari VD (2017). *Lagenaria siceraria* aided green synthesis of ZnO NPs: Anti-dandruff, anti-microbial and anti-arthritic activity. Res. J. Chem. Environ..

[50] Nagarajan KV, Vijayarangan DR (2018). *Lagenaria siceraria*-synthesised ZnO NPs—a valuable green route to control the malaria vector *Anopheles stephensi*. IET Nanobiotechnol..

[51] Ishwarya R (2018). Facile green synthesis of zinc oxide nanoparticles using *Ulva lactuca* seaweed extract and evaluation of their photocatalytic, antibiofilm and insecticidal activity. J. Photochem. Photobiol. B.

[52] Suganya P (2017). Biopolymer zein-coated gold nanoparticles: synthesis, antibacterial potential, toxicity and histopathological effects against the Zika virus vector *Aedes aegypti*. J. Photochem. Photobiol. B.

[53] Ragavendran C, Mariappan T, Natarajan D (2017). Larvicidal, histopathological efficacy of *Penicillium daleae* against larvae of *Culex quinquefasciatus* and *Aedes aegypti* plus biotoxicity on *Artemia nauplii* a non-target aquatic organism. Front. Pharmacol..

[54] Banumathi B (2017). *Euphorbia rothiana*-fabricated Ag nanoparticles showed high toxicity on *Aedes aegypti* larvae and growth inhibition on microbial pathogens: A focus on morphological changes in Mosquitoes and Antibiofilm potential against Bacteria. J. Cluster Sci..

[55] Chandramohan B (2016). Characterization and mosquitocidal potential of neem cake-synthesized silver nanoparticles: Genotoxicity and impact on predation efficiency of mosquito natural enemies. Parasitol. Res..

[56] Murugan K (2015). *Cymbopogon citratus*-synthesized gold nanoparticles boost the predation efficiency of copepod *Mesocyclops aspericornis* against malaria and dengue mosquitoes. Exp. Parasitol..

[57] Srivastava K, Puri S (2004). *Plasmodium falciparum*: Modified medium composition supports continuous cultivation with foetal bovine serum. Exp. Parasitol..

[58] Singh S, Srivastava RK, Srivastava M, Puri S, Srivastava K (2011). In-vitro culture of *Plasmodium falciparum*: utility of modified (RPNI) medium for drug-sensitivity studies using SYBR Green I assay. Exp. Parasitol..

[59] Afshar FH (2011). Evaluation of antimalarial, free-radical-scavenging and insecticidal activities of *Artemisia scoparia* and *A. spicigera*. Asteraceae. Revista Brasileira de Farmacognosia.

[60] Bhuvaneswari R, Xavier RJ, Arumugam M (2016). Larvicidal property of green synthesized silver nanoparticles against vector mosquitoes (*Anopheles stephensi* and Aedes aegypti). J. King Saud Univ. Sci..

[61] Shanmugasundaram T, Balagurunathan R (2015). Mosquito larvicidal activity of silver nanoparticles synthesised using actinobacterium, Streptomyces sp. M25 against *Anopheles subpictus*, *Culex quinquefasciatus* and *Aedes aegypti*. J. Parasitic Dis..

[62] Subramaniam J (2016). Multipurpose effectiveness of *Couroupita guianensis*-synthesized gold nanoparticles: High antiplasmodial potential, field efficacy against malaria vectors and synergy with *Aplocheilus lineatus* predators. Environ. Sci. Pollut. Res..

[63] Haldar KM, Haldar B, Chandra G (2013). Fabrication, characterization and mosquito larvicidal bioassay of silver nanoparticles synthesized from aqueous fruit extract of putranjiva, *Drypetes roxburghii* (Wall.). Parasitol. Res..

[64] Benelli G (2015). Research in mosquito control: current challenges for a brighter future. Parasitol. Res..

[65] Kulkarni BD (2016). In vitro cytotoxicity studies of Zn (Zinc) nanoparticles synthesized from *Abutilon indicum* L. against human cervical cancer (HeLa) cell lines. Pharmacogn. J..

